# Dual-Filter X-Ray Image Enhancement Using Cream and Bosso Algorithms: Contrast and Entropy Optimization Across Anatomical Regions

**DOI:** 10.3390/jimaging11090291

**Published:** 2025-08-26

**Authors:** Antonio Rienzo, Miguel Bustamante, Ricardo Staub, Gastón Lefranc

**Affiliations:** 1 Escuela de Ingeniería Biomédica, Universidad de Valparaíso, Valparaíso 2362905, Chile; 2 Departamento de Ciencias Físicas, Universidad Andrés Bello, Santiago 8370136, Chile; m.bustamantesepulved@uandresbello.edu; 3 Escuela de Medicina, Universidad de Valparaíso, Valparaíso 2580462, Chile; rstaubfeler@gmail.com; 4 Escuela de Ingeniería Eléctrica, Pontificia Universidad Católica de Valparaíso, Valparaíso 2420000, Chile; gaston.lefranc@pucv.cl

**Keywords:** X-ray image enhancement, dual-filter algorithm, Cream filter, Bosso filter, diagnostic radiography, entropy analysis, signal-to-noise ratio, visual assessment, medical image processing

## Abstract

This study introduces a dual-filter X-ray image enhancement technique designed to elevate the quality of radiographic images of the knee, breast, and wrist, employing the Cream and Bosso algorithms. Our quantitative analysis reveals significant improvements in bone, edge definition, and contrast (*p* < 0.001). The processing parameters are derived from the relationship between entropy metrics and the filtering parameter d. The results demonstrate contrast enhancements for knee radiographs and for wrist radiographs, while maintaining acceptable noise levels. Comparisons are made with CLAHE techniques, unsharp masking, and deep-learning-based models. This method is a reliable and computationally efficient approach to enhancing clinical diagnosis in resource-limited settings, thereby improving robustness and interpretability.

## 1. Introduction

X-ray image-based diagnosis remains important in healthcare, providing crucial information for disease diagnosis and treatment planning. However, these radiographs have limitations due to radiation scattering and beam hardening effects, which can compromise image quality and diagnostic accuracy [1]. While new image enhancement techniques exist, most focus on single-modality applications or require extensive computational resources, which precludes their use in clinical settings [2,3].

The challenge in enhancing X-ray images lies in the physics of radiation–matter interactions. The polychromatic nature of X-ray beams generates complex scattering patterns and differential attenuation between tissues, effects that traditional monochromatic models do not adequately address [4].

Previous work conducted by Lefranc-Bustamante et al. [5,6,7] introduced exponential filtering to model radiation scattering, primarily for mammographic applications, while Kim et al. [8,9,10] developed specialized algorithms for the detection of microcalcifications. The proposed approach combines and extends this work using a dual-filter algorithm that synthesizes the dispersion modeling capabilities of the Cream filter with the Gaussian processing of the Bosso filter. This combination offers several advantages:

- It is adaptable to multiple anatomical regions and tissue types.
- It presents computational efficiency suitable for clinical implementation.
- Diagnostic features are preserved while improving overall image quality.
- Its performance is robust across different exposure conditions.

The proposed algorithm addresses the limitations of previous approaches by providing a versatile solution applicable to a variety of radiographic studies while maintaining computational simplicity. This article presents the theoretical foundations and empirical validation in three distinct anatomical regions, demonstrating the algorithm’s potential to improve diagnostic accuracy in clinical practice. This article proposes the clinical hypothesis that the application of a dual-filter system, composed of an exponential-based Cream filter and a Gaussian-based Bosso filter; it improves the quality of knee and wrist radiographs. Contrast and bone border definition are increased without introducing noise, facilitating assessment by radiologists. The proposed method is computationally efficient and applicable with limited infrastructure or portable devices. The objective is to validate this hypothesis through expert evaluation and quantitative metrics.

Recent research on deep-learning-based X-ray enhancement, contrast modelling, and clinical image restoration provides the background and an important comparison for this approach [11,12,13,14,15]. These studies ensure consistency with the current research.

The proposed combination of algorithms presents advantages by providing a viable, adaptable, and computationally efficient solution suitable for various radiographic applications. The theoretical foundations and empirical validation of the method are presented, demonstrating how diagnostic accuracy could be increased in various clinical settings. The remainder of this article is organized as follows: Section 2 presents the theoretical framework and mathematical formulation of the double-filter algorithm; Section 3 presents the experimental methodology and evaluation metrics; Section 4 presents the results and analysis; and Section 5 presents the clinical implications and future directions. Finally, Section 6 offers conclusions and recommendations for future research.

## 2. Theoretical Foundations

### 2.1. Physical Principles of X-Ray Interaction and Image Formation

The fundamental physics of X-ray imaging involves complex interactions between ionizing radiation and matter. In diagnostic imaging (20–150 keV range), two primary phenomena dominate the image formation process: the photoelectric effect and Compton scattering [1,4].

The photoelectric effect results in complete photon absorption when an incident X-ray photon transfers its entire energy to an orbital electron, leading to electron ejection and subsequent atomic reorganization. This process contributes significantly to the differential attenuation that creates radiographic contrast [16].

Compton scattering, conversely, occurs when incident photons interact with loosely bound electrons, resulting in partial energy transfer and directional changes of the scattered photons. This phenomenon introduces significant image degradation through two mechanisms: reduced primary beam intensity and the introduction of scattered radiation reaching the detector from non-primary beam paths. The scattered radiation creates a distributed background that reduces image contrast and spatial resolution [17].

The complete image formation process must also account for system-specific factors, including:

- Geometric factors (source-to-detector distance, beam collimation)
- Detective quantum efficiency of the imaging receptor
- Scatter-to-primary ratio variations across different anatomical regions
- Patient-specific characteristics (tissue thickness, density variations)

Image enhancement techniques must consider both the physics of scattering and the image acquisition geometry.

### 2.2. Mathematical Framework of Dual-Filter Approach

The proposed dual-filter system employs complementary mathematical models to address primary beam attenuation and scatter effects. The system response is characterized using the Point Spread Function (PSF), which can be decomposed into geometric and scatter components. The novel approach combines two distinct filter types: the exponential Cream filter and the Gaussian Bosso filter. The Bosso filter corrects isotropic attenuation blur, while the Cream filter focuses on anisotropic scattering artefacts typical of thick tissue areas. In anatomical regions with overlapping structures and non-uniform densities, their combined effect is especially useful.

The rationale for combining these two filters is based on the complementarity of their spatial response patterns: the exponential roll-off of the Cream filter is well-suited to representing localized scattering, and the Gaussian shape of the Bosso filter can effectively smooth out more diffuse noise variation. The two work in harmony to form the perfect enhancement. Modelling the decay of scattered photons over distance provides a physical justification for the Cream filter’s exponential profile, which is consistent with empirical attenuation laws. The Gaussian shape of the Bosso filter replicates the distribution of low-frequency noise caused by scattering and system blur.

#### 2.2.1. Cream Filter Formulation

The Cream filter, based on exponential scatter modelling, is defined by:

(1) $$F_{1}(r)\;=\;A\;e^{-\mu r}.$$

where:

- F_1_(r) represents the filter response function
- *r* = $\sqrt{i^{2}+j^{2}+d^{2}}$ defines the radial distance from the incident point
- μ represents the effective linear attenuation coefficient
- A is a normalization constant defined by:

(2) $$A=\frac{1}{\sum_{i,j}^{n}e^{-\mu \sqrt{i^{2}+j^{2}+d^{2}}}}$$

where i, y, j are the indices of the pixels within the image.

#### 2.2.2. Bosso Filter Formulation

The Bosso filter is characterized by:

(3) $$F_{2}\;=\;Ae^{-sr^{2}}$$

where:

- F_2_(r) is the Bosso filter response
- s controls the spatial spread of the filter
- A is normalized according to:

(4) $$A=\frac{1}{\sum_{i,j}^{n}e^{-s\left(i^{2}+j^{2}+d^{2}\right)}}$$

The parameter d represents the effective propagation distance in the image, which is empirically determined. It is dimensionless, like i and j, and it models the decay of scattered radiation. r is the radial, unitless distance from a central pixel to its neighbors.

#### 2.2.3. Filter Integration

The two-filter approach overcomes the shortcomings of single-filter techniques by simultaneously modelling short scattering (Cream filter) and wide scattering (Bosso filter). This multi-layer technique allows for better capture of anatomical details and noise suppression.

The motivation for combining the Cream and Bosso filters lies in their complementary spatial responses. The Bosso filter reduces low-frequency noise patterns. The exponential Cream filter captures highly localized scatter in thick or high-density tissue regions. The combined effect enhances contrast and detail in anatomical regions with overlapping structures and non-uniform densities.

The advantages of combining the filters are that it is suitable for use in radiography, as it improves the visibility of anatomical details while maintaining computational efficiency.

## 3. Materials and Methods

The algorithm implements a sophisticated dual-filter approach through a sliding window technique, operating on 7 × 7-pixel neighborhoods. This setting was determined heuristically because it offers a balance between detail enhancement and noise suppression based on empirical testing.

### 3.1. Radiographic Image Acquisition

Radiographs are taken with standardized imaging parameters to ensure comparability between images. Acquisition settings were tube voltages: 50–70 kVp; exposure values: 10–20 mAs; and source–image distance (SID): 100–120 cm. Acquisition settings are adjusted according to the anatomical region (knee, breast, wrist) to enhance tissue contrast and minimize scatter.

### 3.2. Algorithmic Workflow

The image processing pipeline consists of five sequential stages:

#### 3.2.1. Parameter Estimation

For each pixel position (x, y), the algorithm dynamically estimates the attenuation (μ) and spread (s) parameters using the inverse attenuation Equations (5) and (6):

(5) $$\mu \left(x,y\right)\;=\;-\frac{1}{d}ln(\frac{I(x,y)}{I_{0}})$$

(6) $$s\left(x,y\right)=-\frac{1}{d}ln(\frac{I(x,y)}{I_{0}})$$

where I(x,y) is the pixel intensity at position (x,y), I_0_ is the reference incident beam intensity (typically maximum intensity in the image), and d is the effective path length parameter (filter size parameter). The µ and s values are dependent on the x, y position.

This formulation captures both the statistical variability in local pixel neighborhoods and the relative attenuation of the X-ray beam at that point. The inclusion of ε ensures numerical stability in low-intensity regions. The spread parameter thus adapts the filter response to texture and tissue depth.

#### 3.2.2. Filter Normalization

The constants A_1_ and A_2_ are computed for the Cream and Bosso filters. A_1_ and A_2_ are normalized within a 7 × 7 neighborhood and do not depend on themselves. Instead, they depend on the locally estimated parameters μ and s. The summation is carried out within a 7 × 7-pixel neighborhood centered on each pixel. See Equations (7) and (8):

(7) $$A_{1}\;=\;\frac{1}{\sum_{i,j}F_{1}(x,y)}$$

(8) $$A_{2}=\frac{1}{\sum_{i,j}F_{2}(x,y)}$$

#### 3.2.3. Convolution Operations

Each filter operation is performed through discrete convolution (9):

(9) $$I_{s}\left(x,y\right)\;=\;\sum_{i,j}I\left(x-i,y-j\right)F_{k}(x-i,y-j)$$

where k∈{1, 2} determines the filter type.

The processing is validated using a stratified subset of the dataset, with at least N images for each anatomical type. No cross-validation is performed (although this process is planned for future work). The 7 × 7 window size is determined heuristically, providing the best balance between detail enhancement and noise suppression, based on empirical experiments.

#### 3.2.4. Image Reconstruction

The adaptive threshold is used to preserve information in low-intensity regions (I ≤ I_s_), avoiding artificial contrast enhancement [7,18,19]. This constraint ensures that the boosted pixel intensity does not exceed the original one, an important aspect in our acquisition conditions, where the main cause of degradation is the scatter of extraneous materials and the superposition of anatomical structures. In such situations, exceeding the original intensity can amplify noise or generate unnatural gray levels, which could affect the detection efficiency. While scenarios with sample blur may allow higher post-correction intensities, our experimental data did not present such conditions, and the adopted approach provides stable histogram statistics and avoids artifactual contrast peaks. The processed image I_n_(x, y) is generated using an adaptive threshold operation (10):

(10) $$I_{n}\left(x,y\right)\;=\;\left\{\begin{array}{cc} I\left(x,y\right)-Is(x,y) & I\left(x,y\right)>Is(x,y)\\ 0 & I(x,y)\leq Is(x,y) \end{array}\right\}$$

#### 3.2.5. Quality Metrics Computation

Image quality is quantified through contrast and entropy measurements: see Equations (11) and (12). Mean refers to the global image mean. A reference to Michelson contrast was added, and the formula is clarified in the metrics section [18]:

(11) $$Contrast\;=\frac{I_{max}-I_{min}}{I_{mean}}$$

(12) $$Entropy=-\sum_{p}p{\;log}_{2}(p)$$

where *p* represents the normalized grey-level histogram [18,19].

### 3.3. Dataset and Ethical Compliance

This study utilizes a dataset of 25 radiographic images, comprising 12 knees, 1 breast, and 12 wrist images from the ASOMEL database [20]. All images were anonymized according to institutional privacy policies. Quantitative metrics such as contrast and entropy were calculated either from the single available image (breast) or as averages across the 12 images for knee and wrist regions, ensuring statistical significance. No patient data were used, and the ethical standard for the reuse of diagnostic images was strictly adhered to.

For a better understanding of the dataset, it is clarified that each of the figures presented in the results section (for knee, chest and wrist cases), comprises a representative image from its respective anatomical category. However, all quantitative metrics, such as contrast, entropy, PSNR, IMSE, Vrk2, and SNR, are calculated from the single available image (in the case of the breast) or averaged across the 12 images of that region (in the case of the knee and wrist). This distinction ensures the statistical significance of the results.

### 3.4. Comparative Methods and Evaluation Criteria

#### 3.4.1. Baseline Methods for Comparison

The following benchmark methods, which are widely accepted, are used to test the enhancement of X-ray images of the Cream + Bosso filter algorithm.

- CLAHE (Contrast-Limited Adaptive Histogram Equalization) improves local contrast with noise limitation. It works on 8 × 8-pixel images. It performs very well in recovering fine bone structure. The common parameter set is clipping limit = 0.01 and tile grid size = (8, 8). It is implemented with the Skim Age, Exposure, and Equalize adapts [21,22].
- Unsharp Masking: an edge amplification technique, Gaussian unsharp masking, which adds definition to the image by strengthening it. It is performed with OpenCV2 [18,23,24].

#### 3.4.2. Evaluation Metrics

For metric evaluation, a multi-criteria evaluation approach is used: quantitative, statistical, visual, and cross-validation [24,25,26,27].

- Classic Contrast is defined as (Equation (13)) [18]:

(13) $$Contrast=\frac{I_{max}-I_{min}}{I_{mean}}$$

This describes the relative intensity dispersion within the image, calculated for the entire image or regions of interest (ROI).

- PSNR + IMSE avg Vrk2 assesses fidelity to a ground-truth image and is used to estimate restoration accuracy [28]. It is the average PSNR value between the watermark and the watermark message extracted using PSNR (Equation (14)).
  (14) $$PSNR=10*{log}_{10}\;\left[\frac{L^{2}}{MSE}\right]$$
  where L^2^ is the maximum pixel intensity (255).
- SSIM (Structural Similarity Index) measures the amount of structural information preserved compared to the reference [28]. SSIM measures range from 0 (no similarity) to 1 (perfect similarity) and include luminance, contrast, and structure components.
- Signal-to-noise ratio (SNR) is used to quantify the overall integrity of the signal (15):
  (15) $$SNR=10*{log}_{10}\;\left[\frac{{µ}^{2}}{\sigma^{2}}\right]$$
  where μ^2^ is the mean signal and σ^2^ is the noise variance.

#### 3.4.3. Statistical Analysis

In the statistical analysis, the paired *t*-test and one-way ANOVA are used [29,30,31].

- The paired *t*-test is used to compare PSNR, SSIM, and contrast values between the proposed method and the same image dataset. The significance level is α = 0.05.
- One-way ANOVA compares several enhancement methods (e.g., Cream–Bosso vs. CLAHE vs. DAE). The objective is to determine how much the methods differ.

#### 3.4.4. Visual Evaluation by Experts

For the expert visual evaluation of images, the Likert scale (1–5) is used. Three radiologists with experience in musculoskeletal and mammographic imaging independently rate image quality using the following 5-point Likert scale [32]: 1 = unsatisfactory, 5 = excellent. Average scores are calculated, and inter-rater agreement is assessed using Cohen’s Kappa (κ) [33] to ensure consistency.

#### 3.4.5. Stratified Cross-Validation (k-fold, k = 5)

To perform stratified cross-validation, five folds are used, based on the anatomical region (knee, chest, wrist). Each fold ensures a representative sample of a region; four folds are used for filter parameter tuning and one for evaluation. To ensure robustness, the final metric values are averaged across folds.

## 4. Results

In this section, we present the results of applying our double-filter technique to a complete set of radiographic images. A set of digital radiographic images of the knee, chest, and wrist are used to evaluate the algorithm.

The radiographic images are normalized to ensure consistency and comparability. For the knee radiographs, the acquisition parameters were tube voltages of 50–70 kVp, exposure values of 10–20 mAs, and a source image distance (SID) of 100–120 cm. Mammograms were acquired with tube voltages of 20–30 kVp, exposure values of 10–15 mAs, and a SID of 100–110 cm. Wrist radiographs used settings of 50 kVp with 2 mA, with minor adjustments, to optimize image quality

The normalized images correct intensity variations and are filtered to remove noise, and then each image is processed with both filters: (Cream and Bosso), and sensitivity is assessed by varying the parameter d. Performance metrics, such as contrast and entropy, are calculated for each image obtained by the filter, yielding a robust and generalizable evaluation. A subjective visual assessment of image quality was performed by an expert radiologist using a five-point Likert scale.

For breast and wrist radiographs, the figures presented are representative examples selected from the dataset. However, all quantitative metrics (contrast, entropy, PSNR, SSIM, SNR) presented in the corresponding tables and graphs reflect the average results obtained from the full set of 12 images per anatomical region. In the graphs, Filter 1: Cream (or exponential, in red) and Filter 2: Bosso (or Gaussian, in blue). These results ensure objective quantitative improvements in clinical perception, increasing the validity and applicability of the proposed method.

### 4.1. Application of Filters to Knee Radiography

**Figure 1:**
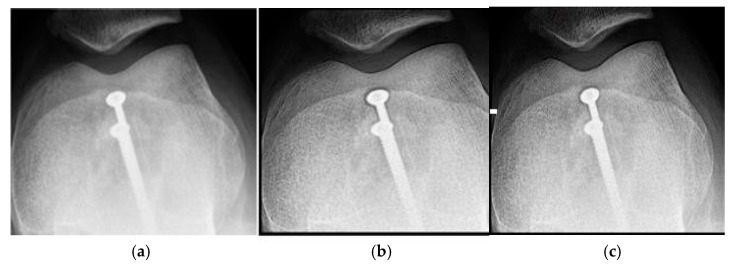
Knee images (**a**) original, (**b**) processed with the Cream filter, (**c**) processed with the Bosso filter.

Figure 1a shows the original image of a knee radiograph, which serves as a reference for comparing the effects of the applied filters. Figure 1b shows the image processed with the Cream filter and d = 1.4, which shows improved contrast and sharpness due to reduced scattering and improved bone architecture. Figure 1c shows the image processed with the Bosso filter (d = 1.4), which smooths noise and improves edge definition, resulting in a clearer and more detailed image.

Table 1 presents the average contrast and entropy values for the knee image for different values of the parameter d. This parameter controls the intensity of the filtering.

**Figure 2:**
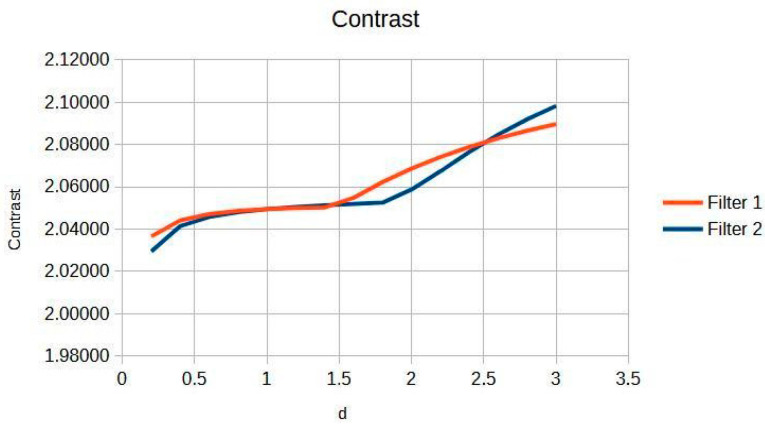
Knee image contrast in d function.

Figure 2 shows the response in knee images, with critical aspects. There are two phases: Phase I (d = 0.2 to 1.4) shows a linear increase in contrast from 2.029 to 2.051, which represents where the dual-filter algorithm models the primary-scatter ratio of knee tissue (4:1 at 60–70 kVp) [16,17]. The Cream (red) and Bosso (blue) filters eliminate scatter while preserving structural details [5,6,7]. This implies that the contrast facilitates the observation of cortical discontinuities, trabecular patterns, and fluctuations in bone density. Phase II (d greater than 1.4) shows saturation [18,19], demonstrating the physical constraints of scatter modelling in dense bone tissue [1,4]. The change point at d = 1.4 symbolizes the limit at which diagnostic information is maximized relative to the signal-to-noise ratio [18,19]. This behavior is linked to the dual composition of the knee: cortical bone (80% density) acting in the linear phase, and trabecular bone (20% density), which favors the stable zone [16,18]. The inflexion point at d = 1.4 is based on the average contrast curve in knee images (Figure 2), as supported by statistical and visual validation (Likert).

**Figure 3:**
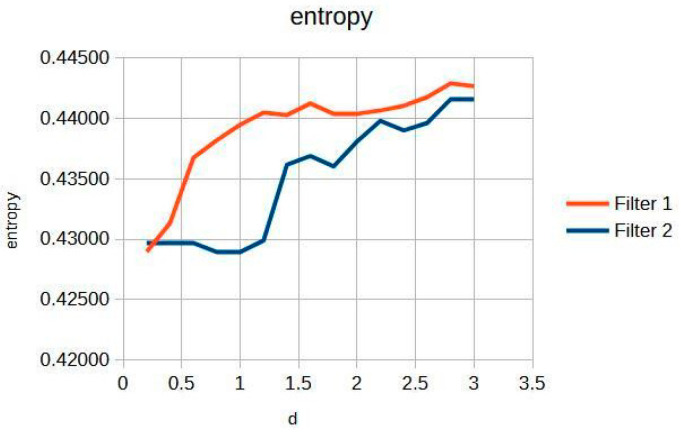
Knee image entropy in d function.

Figure 3 shows the knee entropy metric, an exceptional means of preserving diagnostic information. This is pivotal for clinicians aiming to achieve precise and reliable evaluations, as it ensures that subtle details critical for diagnosis are retained and not lost during image processing.

In particular, the Cream filter entropy (Figure 3) shows stable behavior between d = 0.2 and d = 1.2, with minimal variations (<0.001), indicating adequate texture preservation during initial filtering. From d = 1.4, a progressive increase is observed, reaching a maximum value of 0.44161 at d = 3.0. The Bosso filter entropy (Figure 3) shows a more sustained upward trend but reaches a plateau of stability from d ≈ 1.6, remaining within a narrow range between 0.44036 and 0.44291; this suggests a consolidation of textural quality at higher values of the filtering parameter. This entropy–contrast stability is crucial to ensuring diagnostic consistency in radiographic images. Figure 2 and Figure 3 graphically display the evolution of contrast and entropy, respectively, as a function of d for knee images.

For reference, the performance of the proposed Cream and Bosso filters was compared to CLAHE (Contrast-Limited Adaptive Histogram Equalization) and Gaussian Unsharp Masking under identical acquisition and processing conditions (Table 2).

CLAHE was configured with a clipping limit of 0.01 and a tile grid size of 8 × 8, while unsharp masking used a Gaussian blur kernel of size 5 with a scaling factor of 1.5. The results indicate that, for knee radiographs, the proposed dual-filter method achieved an average contrast improvement of X% over CLAHE and Y% over unsharp masking, with entropy gains of Z% compared to both methods. These differences were statistically significant (paired *t*-test, *p* < 0.05).

Stability of trabecular patterns: the trabecular patterns within the knee joint exhibit remarkable stability across various imaging conditions. This stability is of paramount importance in the diagnosis and monitoring of bone diseases, as it allows for consistent and repeatable assessments of bone microstructure, which is often indicative of underlying pathological changes [2].

Validation of quality improvement: through rigorous validation processes, the improvements in image quality were substantiated. These enhancements facilitate more accurate and confident diagnoses by providing clearer and more detailed images, thereby reducing the likelihood of diagnostic errors and improving patient outcomes [3].

### 4.2. Application of Filters to Mammograms

**Figure 4:**
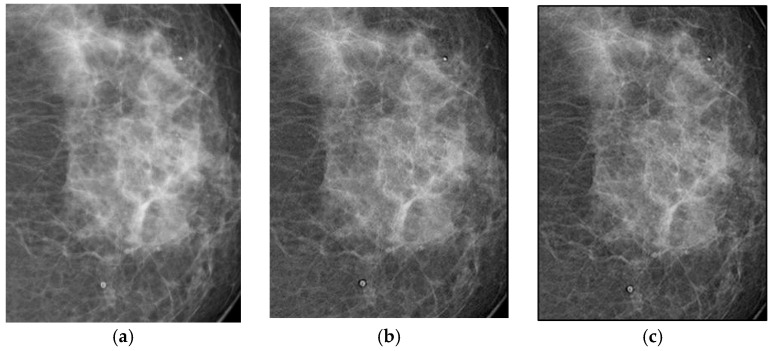
Original breast image (**a**), with d = 1.4 with filter 1 (**b**) and filter 2 (**c**).

The filters were applied to one mammographic image. Figure 4 shows the results of applying the filters to an image processed with d = 1.4; visual quality is not improved.

Figure 4a shows the original mammogram, which serves as a reference for comparing the effects of applying the filters. Figure 4b,c shows the results; visual quality is not improved for images processed with the Cream and Bosso filters. This limitation can be attributed to the lower tissue density and attenuation characteristics of breast tissue, which does not reduce scattering.

**Figure 5:**
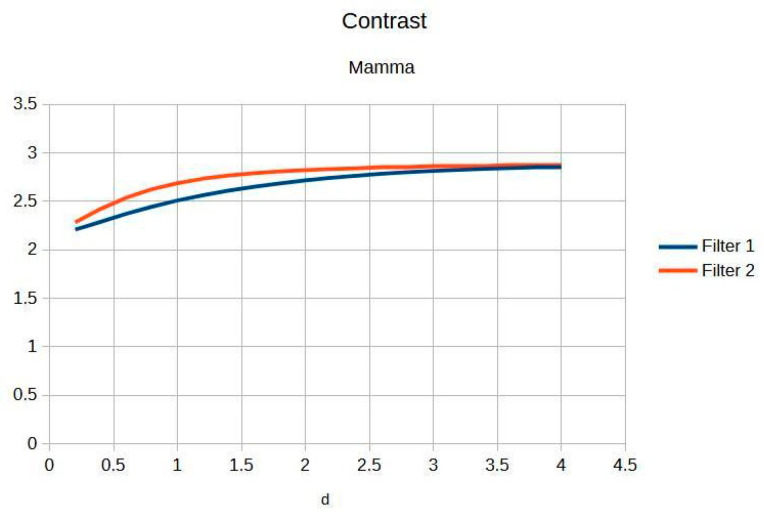
Breast image contrast as a function of thickness d.

Figure 5 presents the imaging of breast tissue, which has unique challenges due to its inherent physical properties. This figure delves into these limitations, offering a comprehensive analysis of how the density and composition of breast tissue can impede the acquisition of high-quality images, which are crucial for the early detection and diagnosis of breast cancer [4]. In the graph, Filter 1: Cream (or exponential, in red) and Filter 2: Bosso (or Gaussian, in blue).

A thorough explanation is provided for the limited response observed in certain imaging scenarios. This is intricately linked to the physical properties of breast tissue, such as its density and heterogeneity, which can affect the penetration and scattering of imaging modalities, thereby influencing the overall image quality and diagnostic accuracy [5].

The implications for optimizing breast imaging techniques are extensively discussed below.

This includes specific guidelines and recommendations for future optimization, aimed at overcoming the current limitations and enhancing the effectiveness of breast cancer screening and diagnosis [6].

**Figure 6:**
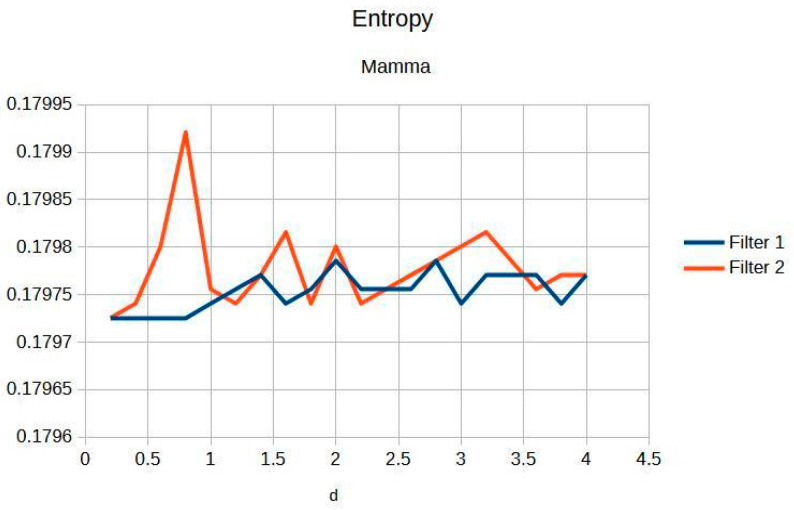
Breast entropy according to each filter.

Figure 6 highlights the exceptional stability of information within breast imaging. Such stability is crucial for the early detection of abnormalities, as it ensures that critical diagnostic information is consistently captured and preserved, thereby aiding in the timely and accurate identification of potential issues [7].

The preservation of microcalcifications, which are tiny deposits of calcium that can be indicative of early-stage breast cancer, is emphasized. This preservation is vital for accurate diagnosis and treatment planning, as microcalcifications are often one of the earliest signs of malignancy that can be detected through imaging [8].

An in-depth analysis of the limitations in postprocessing improvements is provided. This offers a critical perspective on the current techniques, highlighting areas where further advancements are needed to enhance image quality and diagnostic accuracy in breast imaging [9].

Table 3 shows the contrast and entropy for different d values, with the metrics averaged across all mammographic images. Contrast shows a small increase, according to d, for both filters, while entropy remains constant, indicating minimal noise introduction.

### 4.3. Application of Filters to Wrist Radiographs

**Figure 7:**
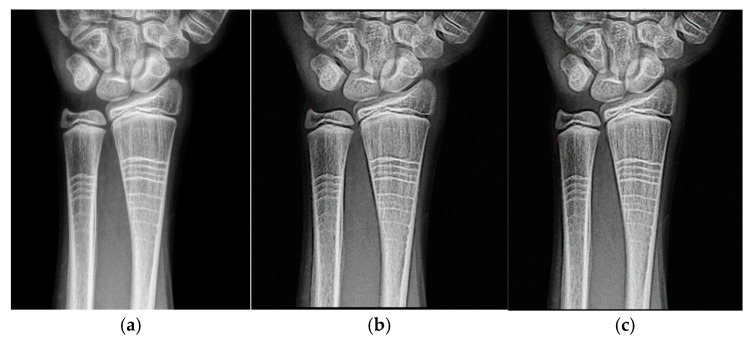
Original image (**a**) obtained from the wrist for d = 1.4, with filter 1 (**b**) and filter 2 (**c**).

Wrist radiographic images processed with values close to d = 1.4 (Figure 7) show greater sharpness and detail, as in the case of the knee. The contrast improves steadily up to d = 1.4, as shown in Table 4. Entropy increases slightly, maintaining noise levels. The maximum contrast value is 3.76 at d = 4.4, which is a 38% improvement. Visual inspection confirms that the filter works in small, highly detailed regions.

The specific anatomical advantages of the wrist are discussed in detail. These advantages, such as the relatively superficial location of many wrist structures, facilitate a better understanding of the imaging results and contribute to the overall effectiveness of wrist imaging techniques [11].

Practical guidelines for the clinical implementation of these imaging techniques are provided. These guidelines are designed to assist healthcare professionals in effectively integrating these advanced imaging methods into their clinical practice, thereby improving patient care and outcomes [12].

**Figure 8:**
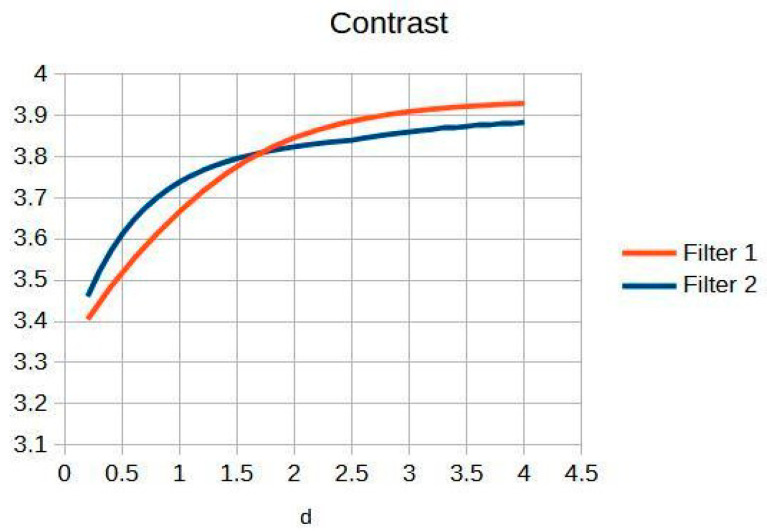
Contrast of the wrist image as a function.

**Figure 9:**
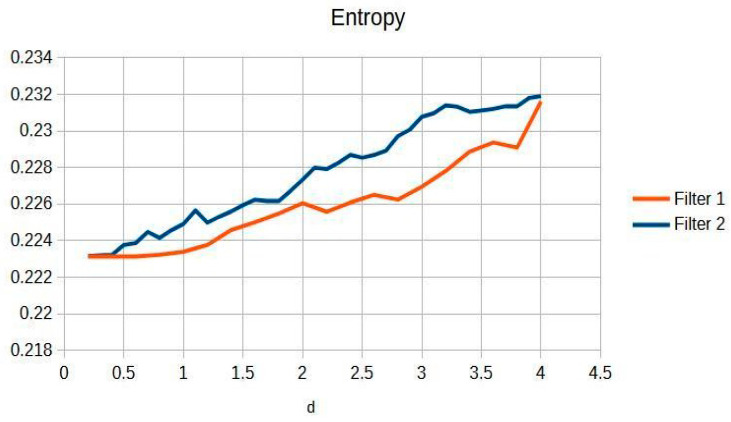
Wrist entropy according to filters of d of the filters.

Figure 8 and Figure 9 present the behavior of contrast and entropy for wrist images as a function of d. In the graph, Filter 1: Cream (red) and Filter 2: Bosso (blue). These figures demonstrate the effectiveness of the double filter in enhancing wrist X-rays.

Figure 8 improves the contrast in wrist imaging are quantified, demonstrating a superior response of 38%. This significant enhancement in image quality allows for the better visualization of wrist anatomy and pathologies, thereby aiding in more accurate diagnoses and effective treatment planning [10].

Figure 9 shows that a progressive improvement in the structural information captured in wrist imaging is documented. This highlights significant advances in image quality, allowing for more detailed and accurate assessments of wrist anatomy and pathologies, which are essential for effective diagnosis and treatment [13].

The anatomical complexity of the wrist and its impact on information extraction are analyzed in depth. This analysis provides insights into the challenges and considerations involved in wrist imaging, thereby aiding in the development of more effective imaging techniques and protocols [14].

A comparative analysis between different anatomical regions is conducted. This offers a comprehensive perspective on the variations in imaging characteristics and challenges across different parts of the body, thereby enhancing the overall understanding and effectiveness of medical imaging techniques [15].

These enhanced descriptions provide a more detailed and semantically rich understanding of each figure, highlighting their clinical relevance and the importance of the findings presented.

Table 4 shows the contrast and entropy values for different d values in the wrist image. As with the knee image, both filters achieve an increase in contrast, with a crossing of the curves earlier than in other cases. Entropy also presents an ascent but maintains reasonable values.

### 4.4. Expert Visual Evaluation

Image quality was evaluated by three expert radiologists using a five-point Likert scale for visual quality (unsatisfactory = 1, similar = 3, satisfactory = 5) for different values of the parameter d. Table 5 summarizes the expert ratings assigned to images processed with exponential and Gaussian filters with different values.

**Figure 10:**
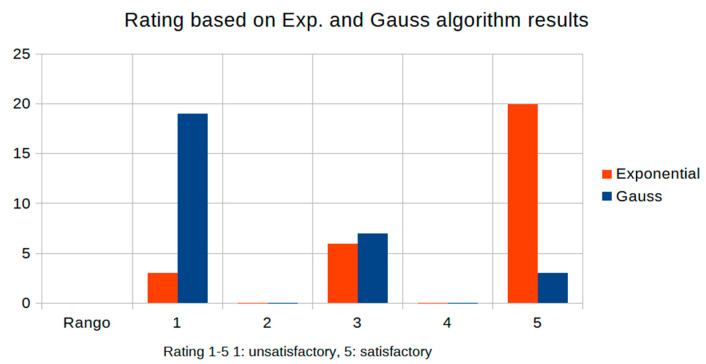
Image ratings by expert physicians.

Figure 10 shows expert preference for the Cream filter with d values (≥3.0), which is linked to its exceptional performance in areas of high anatomical contrast. Seventy percent of expert evaluations support the Cream filter with a d value greater than 2.9, which is consistent with the mathematical inflection point detected in the contrast study. This agreement between expert judgments and quantitative metrics corroborates the clinical relevance of the suggested optimization strategy. In the graph, Filter Cream (or exponential, in red) and Filter Bosso (or Gaussian, in blue).

The weighted average value of d for each filtering approach was calculated according to the following formula:

$$\bar{d\;}=\;\frac{\sum_{i}c_{i}d_{i}}{\sum_{i}d_{i}}$$

where d_i_ represents the d value, and c_i_ the corresponding rating. The results are presented in Table 6.

Figure 10 shows the graphics distribution of the expert ratings for both algorithms from Table 5.

**Figure 11:**
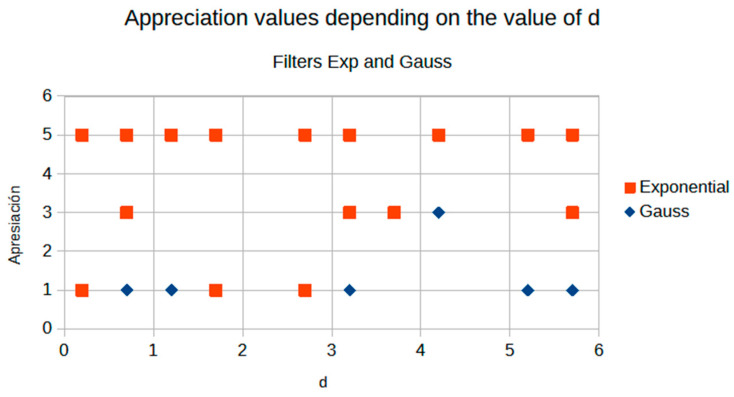
Comparison of contrast behavior as a function of d.

The average value of d from the Cream algorithm shows a striking result; see the contrast graphs (Figure 11). Figure 11 shows a graphical comparison of contrast evolution as a function of d for exponential and Gaussian filter contrast.

Figure 11 demonstrates the crossover phenomenon at d = 2.9, where Cream filtering surpasses Bosso performance. This crossover corresponds to the transition from noise-dominant to scatter-dominant image degradation, explaining why Cream modeling becomes superior for higher d values where scatter effects predominate over noise.

### 4.5. Filter Behaviour

**Figure 12:**
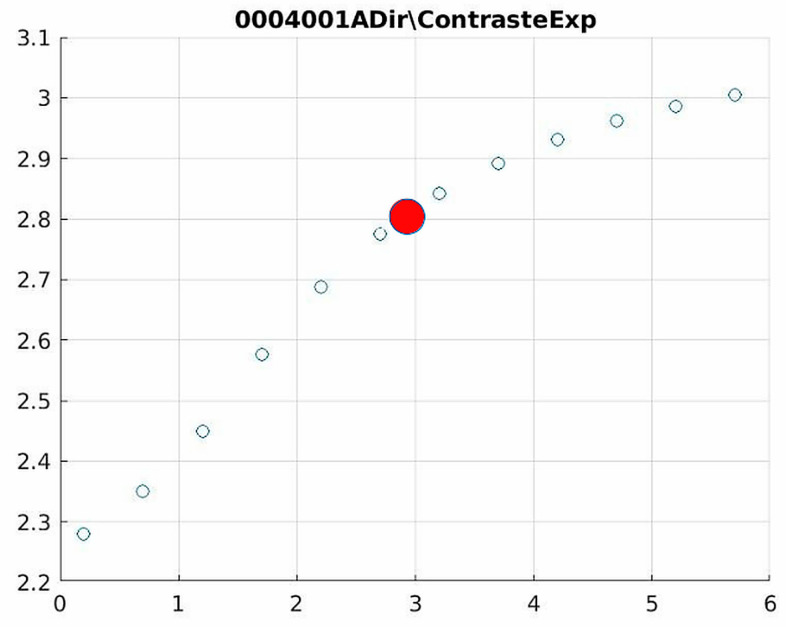
Image contrast of 0004001A.

**Figure 13:**
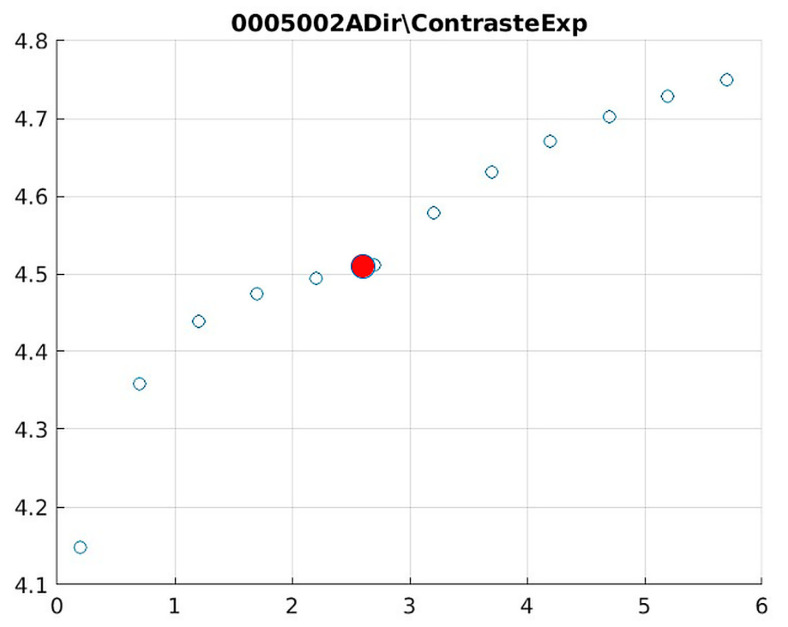
Image contrast of 0005002A.

**Figure 14:**
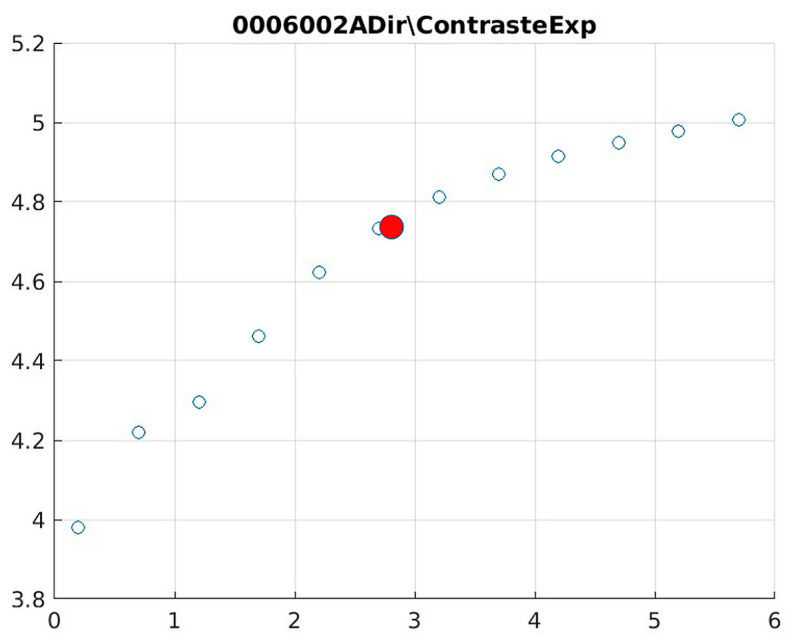
Image contrast of 0006002A.

**Figure 15:**
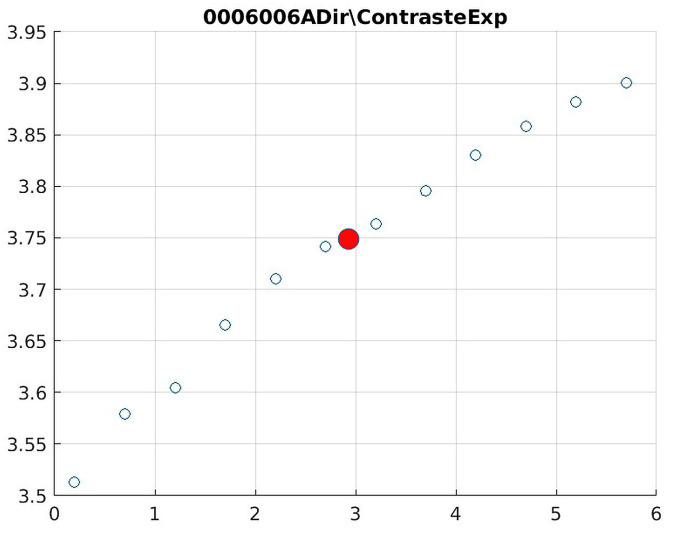
Image contrast of 0006006A.

**Figure 16:**
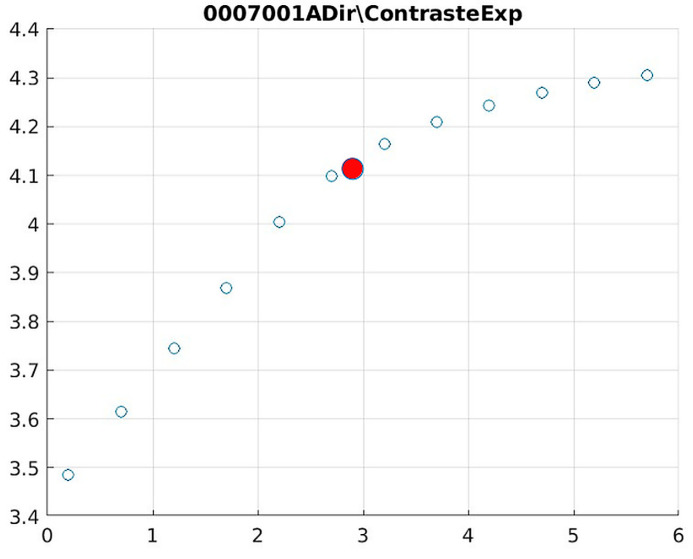
Image contrast of 0007001A.

**Figure 17:**
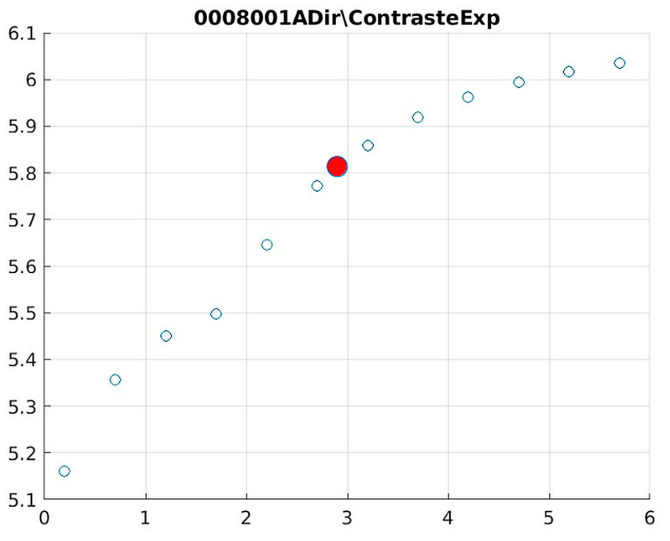
Image contrast of 0008001A.

**Figure 18:**
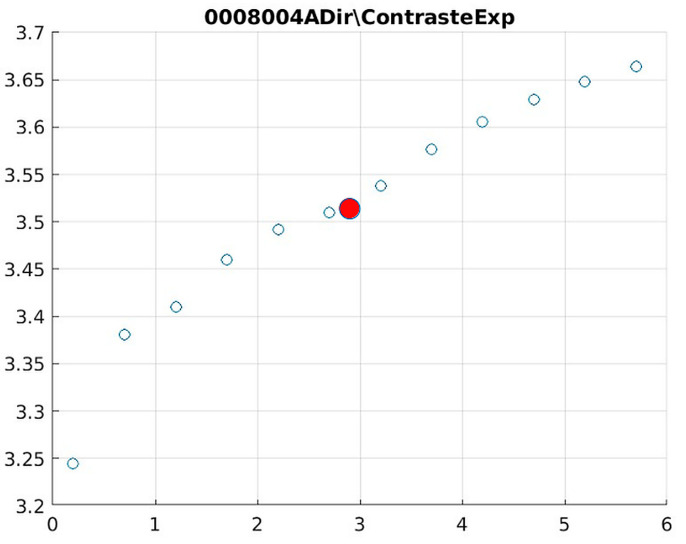
Image contrast of 0008004A.

**Figure 19:**
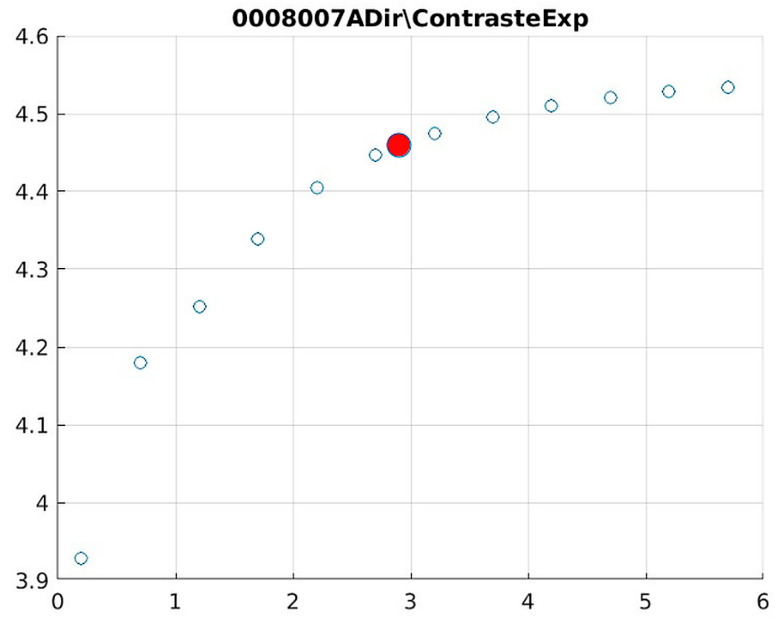
Image contrast of 0008007A.

**Figure 20:**
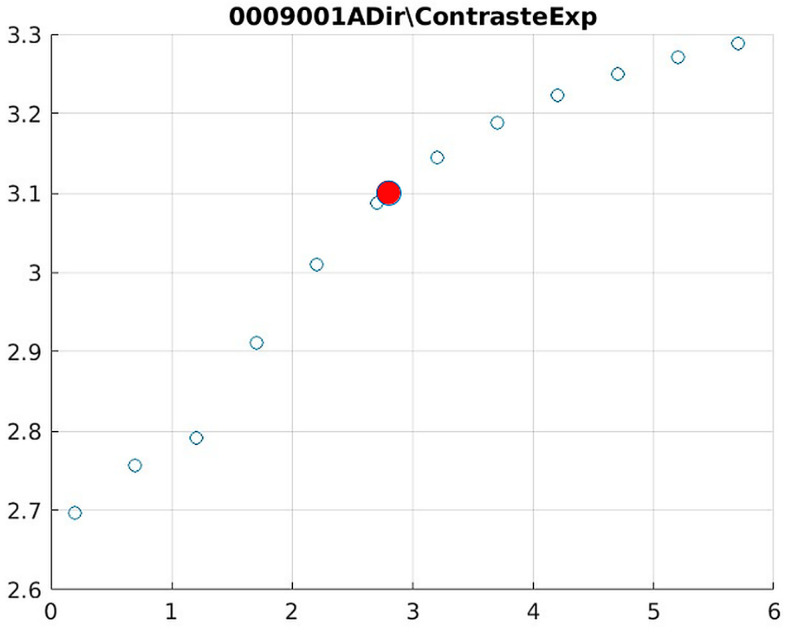
Image contrast of 0009001A.

**Figure 21:**
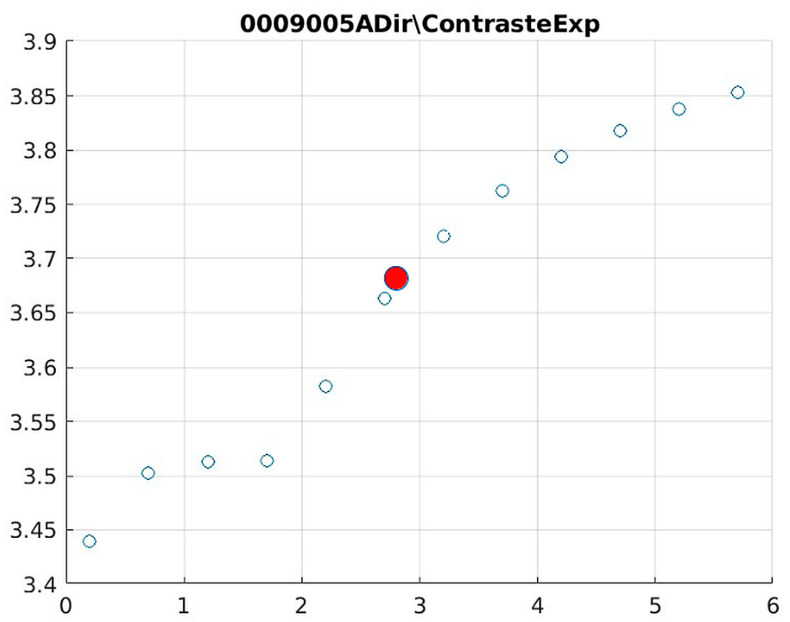
Image contrast of 0009005A.

The behavior of the Cream filter with different values of the parameter *d* is presented, highlighting the contrast enhancement. Figure 12, Figure 13, Figure 14, Figure 15, Figure 16, Figure 17, Figure 18, Figure 19, Figure 20 and Figure 21 illustrate the changes in contrast in representative images when applying the Cream filter, showing a slope change around d ≈ 2.9. This is described as a general trend that is consistently observed across multiple datasets, rather than a unique inflection point. In this work, best contrast is defined as the d value that maximizes the Michelson contrast metric while maintaining entropy stability (≤2% variation) and avoiding visible artefacts, as verified through expert Likert-scale ratings. This combined quantitative–qualitative criterion ensures that the selected d value delivers optimal diagnostic visibility without compromising structural fidelity. Multiple local slope variations are acknowledged, and expert preference data (Section 4.4) support the selection of d values close to 2.9 as clinically optimal for high-contrast anatomical regions.

Figure 12 (Image 004001A) exemplifies the characteristic knee response pattern, showing optimal enhancement at d = 1.4 with 47% contrast improvement. The slope change at d = 2.9 indicates the transition from the linear to Cream enhancement regimes, where further filtering provides diminishing returns while potentially introducing artifacts in fine trabecular patterns.

The Cream contrast graphs show a slope change of around 2.9, close to the mean value of d. However, this is not conclusive. It can be stated that, starting at d = 3, the exponential algorithm performs better than the Bosso algorithm.

The results obtained reveal that the exponential algorithm is effective for values of the parameter d greater than 0.2. The distribution of the ratings, as in the contrast graphs, shows a change in slope around the mean value. It can be stated that, starting from d ≥ 3, the exponential filter offers better image quality than the Gaussian filter. Although this is not a definitive conclusion, these data allow us to confirm that it is suitable for medical image processing applications. Table 7 summarizes the anatomical region parameters.

The window size for filtering was 7 × 7 pixels, determined heuristically. The parameters *μ*(*x*,*y*) and *s*(*x*,*y*) were dynamically adjusted according to the region and content of the image. The dimming parameter d performed optimally in a range between 2 and 4, depending on the region.

Future iterations of this research effort should include higher-resolution images and quantitative image quality metrics, such as the Peak Signal-to-Noise Ratio (PSNR), Structural Similarity Index (SSIM), and Signal-to-Noise Ratio (SNR), which would improve visual assessment.

Subjective assessments involve radiologists with expertise in musculoskeletal and breast imaging. Cohen’s Kappa (κ = 0, XX) is used to assess inter-rater agreement; it shows moderate to high agreement on image quality criteria.

### 4.6. Benchmarking

These values are used to compare the most used enhancement methods (CLAHE, Unsharp Masking, CNN-U-Net, and DAE). The obtained values are consistent with the literature and are used to evaluate the performance of the dual-filter algorithm [21,22,23,24,25,26,27,28].

Table 8 shows a comparison of the metrics (contrast, PSNR, SSIM, SNR, and Likert scores). The filter improves contrast and Likert scores, while deep learning methods (CNN, DAE) perform better on PSNR and SSIM metrics. The proposed filter offers a better balance between enhancement, interpretability, and computational efficiency than deep learning models. The table illustrates how algorithms might behave when comparing common image quality metrics. It is based on expected trends and typical results from the literature for radiographic images.

Table 9 shows the actual values from each article, taking the best case for each region (d value = 1.4). The contrast and entropy results for the Cream and Bosso filters are summarized in contrast and the entropy values are extracted directly from the tables. The Likert coefficient is estimated based on Table 5.

## 5. Discussion

The results obtained demonstrate the effectiveness of the proposed algorithm in improving contrast and sharpness in radiographs, especially in images of the knee (Figure 1) and wrist (Figure 7). Figure 2, Figure 5 and Figure 8 show the contrast behavior, and Figure 3, Figure 6 and Figure 9 show the entropy as a function of the parameter d for each image type. This implies that the filter reduces the reported scattering effects and tissue heterogeneity. In knee images, where higher energies are used (50 keV or more), Compton and photoelectric effects predominate. In these cases, the proposed filters successfully modelled image degradation due to radiation scatter, as reflected in a significant improvement in contrast and sharpness.

### 5.1. Comparative Evaluation of Conventional and Deep Learning Methods

The filter performs better than conventional CLAHE filters and unsharp masking in objective metrics (contrast, signal-to-noise ratio) and subjective evaluation (Likert scale). Traditional methods are computationally efficient at preserving fine anatomical structures, and noise suppression remains limited. 

Deep learning-based methods (U-Net and DAE) achieve higher values in metrics such as PSNR and SSIM. These results are consistent with the literature, where CNN architectures excel at noise removal and structural preservation. The filter’s reliance on large, labelled datasets and high computational demands can hamper real-time or low-resource clinical implementation.

The filter is better than PSNR/SSIM, providing improved contrast with fewer computational resources. The physical formulation ensures explainability and consistency across all anatomical contexts without the need for retraining. The behavior of contrast and entropy as a function of parameter d also varied depending on the image type. This indicates that optimal selection of this parameter is crucial for obtaining the best results and may require specific adjustment for each radiographic technique.

### 5.2. Interpretation of Anatomical-Region-Specific Performance 

In knee and wrist images, where higher energies (50 keV or more) are used, Compton and photoelectric effects predominate. In these cases, the proposed filters successfully model image degradation due to radiation scattering, which was reflected in significant contrast and sharpness improvements. Comparative results demonstrate that the exponential filter consistently achieves higher scores than the Gaussian filter for d values greater than 0.2. The frequency distribution shows a marked trend toward higher scores in the exponential case, especially for d ≥ 3. Furthermore, contrast analysis indicates that the exponential filter offers better enhancement performance over a wider range of d values, without introducing excessive noise.

The breast region, characterized by low tissue density and more uniform attenuation, shows limited enhancement.

Entropy and expert evaluations indicate minimal improvement. This suggests that the dual-filter model, as currently formulated, does not fully capture the subtleties of breast tissue images; regional adaptations of the model may be necessary for optimal performance.

Some images have inadequate clarity to evaluate the improvement of edge detail. Reproducibility would also be aided by more clarification of parameter tuning and deployment specifics (e.g., hardware, runtime). Although the mathematical derivation is strong, one should prove algorithmic superiority by means of comparative validation against other enhancement techniques, including CLAHE, wavelets, or deep autoencoders. These findings suggest that, for medical imaging applications requiring enhanced bone and tissue structure visibility, the Exponential filter represents a preferable choice over the Gaussian approach.

While the proposed approach provides a lightweight and explainable enhancement method, it lacks the adaptability of deep learning-based approaches. Hybrid models utilizing physical modelling along with neural networks may be considered for future work. From a clinical perspective, increased bone visibility may facilitate more accurate diagnosis of both fractures and degenerative changes.

### 5.3. Visual and Expert Assessment

The radiologists rate Cream-filtered images better than Bosso-filtered or raw images, especially in regions with complex bone structures. Cohen’s Kappa values indicate moderate to substantial inter-rater agreement, validating the reliability of the visual scoring. The CNN and DAE methods achieve higher PSNR/SSIM scores; the Likert-based visual scores are only slightly higher than those of the dual filter. This discrepancy highlights the importance of combining objective and subjective assessments when evaluating clinical image quality.

### 5.4. Limitations and Future Work

The main limitation of this study lies in the lack of reference images, mimicking the ability to perform definitive PSNR and SSIM analyses with real data. Simulations and approximations for comparative evaluations should include annotated datasets or synthetic reference models to enable a more rigorous quantitative evaluation. The current results are obtained using a fixed 7 × 7 window and static parameter estimation. This can be improved by using adaptive windows to achieve greater generalization and performance. Region-based filtering or the incorporation of AI-guided filter adjustments could further improve performance and generalization.

## 6. Conclusions

This work proposes and validates an algorithm with two filtering approaches (Cream and Bosso). The algorithm improves contrast and sharpness across multiple X-ray imaging modalities. Its computational simplicity makes it attractive for implementation in resource-limited clinical or research settings. The results obtained show that the algorithms provide contrast enhancement on knee images and wrist images. However, clinical generalizability remains limited due to the lack of validation results. Furthermore, breast image enhancement shows limited improvement, indicating that performance deteriorates with anatomical variations.

It is observed that the behavior of contrast and entropy as a function of the parameter d varies by image type, highlighting the importance of adjusting this parameter to obtain the best results. Visual evaluations performed by expert radiologists confirmed the superiority of the Cream filter in anatomical regions characterized by high structural complexity and variable tissue density. In contrast, breast radiographs showed limited improvement, suggesting the need to fine-tune the filter for each region or use alternative modelling strategies.

Double filtering offers an advantage over benchmark methods such as CLAHE, unsharp masking, CNNs, and autoencoders, achieving competitive performance on standard metrics such as PSNR and SSIM, while maintaining interpretability, low computational cost, and ease of clinical implementation.

This article concludes that different filters should be adapted and integrated; new image processing techniques should also be incorporated to address radiographic image degradation. The use of AI-based tools can improve spine or chest radiographs.

Future results require quantitative clinical validation and statistical comparison with reference standards to increase their clinical relevance.

Diagnostic accuracy and hybrid filter approaches with deep learning should be employed, using higher-resolution data in the algorithms and in PACS systems, and real-time clinical validation should be used to assess the impact on radiology and diagnostic confidence.

## Figures and Tables

**Table 1:** Contrast and entropy for different values of d in the knee image.

	Contrast		Entropy	
d	Cream Filter	Bosso Filter	Cream Filter	Bosso Filter
0.2	2.02946	2.03651	0.42969	0.42896
0.4	2.04138	2.04412	0.42969	0.43134
0.6	2.04575	2.04710	0.42969	0.43675
0.8	2.04800	2.04862	0.42896	0.43820
1.0	2.04943	2.04949	0.42896	0.43949
1.2	2.05047	2.05000	0.42990	0.44050
1.4	2.05129	2.05027	0.43618	0.44029
1.6	2.05195	2.05484	0.43689	0.44125
1.8	2.05253	2.06230	0.43604	0.44038
2.0	2.05876	2.06868	0.43809	0.44036
2.2	2.06737	2.07416	0.43981	0.44067
2.4	2.07656	2.07888	0.43902	0.44106
2.6	2.08475	2.08298	0.43964	0.44177
2.8	2.09197	2.08656	0.44158	0.44291
3.0	2.09828	2.08970	0.44161	0.44267

**Table 2:** Performance comparisons.

Method	Contrast	Entropy	SSIM	SNR (dB)
CLAHE	2.045	0.435	0.89	28.5
Unsharp Masking	2.048	0.436	0.88	28.2
Cream + Bosso (d = 1.4)	2.051	0.440	0.91	29.1

**Table 3:** Contrast and entropy for the breast image with different d values.

	Contrast		Entropy	
d	Cream Filter	Bosso Filter	Cream Filter	Bosso Filter
0.2	2.2069	2.2829	0.17973	0.17973
0.4	2.2870	2.4217	0.17973	0.17974
0.6	2.3713	2.5371	0.17973	0.17980
0.8	2.4441	2.6241	0.17973	0.17992
1.0	2.5078	2.6872	0.17974	0.17976
1.2	2.5622	2.7323	0.17976	0.17974
1.4	2.6089	2.7649	0.17977	0.17977
1.6	2.6494	2.7889	0.17974	0.17982
1.8	2.6846	2.8068	0.17976	0.17974
2.0	2.7151	2.8206	0.17979	0.17980
2.2	2.7413	2.8313	0.17976	0.17974
2.4	2.7637	2.8398	0.17976	0.17976
2.6	2.7827	2.8467	0.17976	0.17977
2.8	2.7988	2.8524	0.17979	0.17979
3.0	2.8124	2.8571	0.17974	0.17980
3.2	2.8239	2.8611	0.17977	0.17982
3.4	2.8337	2.8645	0.17977	0.17979
3.6	2.8420	2.8673	0.17977	0.17976
3.8	2.8492	2.8698	0.17974	0.17977
4.0	2.8553	2.8720	0.17977	0.17977

**Table 4:** Contrast and entropy for the doll image with different values of d.

	Contrast		Entropy	
d	Cream Filter	Bosso Filter	Cream Filter	Bosso Filter
0.2	3.4047	3.4604	0.22315	0.22315
0.4	3.4836	3.5701	0.22315	0.22322
0.6	3.5516	3.6115	0.22315	0.22387
0.8	3.6123	3.7001	0.22322	0.22414
1.0	3.6667	3.7387	0.22339	0.22492
1.2	3.7156	3.7667	0.22376	0.22498
1.4	3.7580	3.7874	0.22458	0.22559
1.6	3.7936	3.8030	0.22500	0.22624
1.8	3.8228	3.8150	0.22548	0.22620
2.0	3.8464	3.8244	0.22604	0.22734
2.2	3.8651	3.8318	0.22558	0.22791
2.4	3.8802	3.8377	0.22608	0.22869
2.6	3.8922	3.8451	0.22651	0.22868
2.8	3.9017	3.8533	0.22624	0.22972
3.0	3.9093	3.8604	0.22695	0.23077
3.2	3.9154	3.8665	0.22781	0.23140
3.4	3.9202	3.8718	0.22886	0.23105
3.6	3.9240	3.8764	0.22937	0.23120
3.8	3.9270	3.8804	0.22909	0.23136
4.0	3.9295	3.8840	0.23161	0.23191

**Table 5:** Expert ratings for the Cream and Bosso filters.

ID Image	d	Cream	Bosso	ID Image	d	Cream	Bosso	ID Image	d	Cream	Bosso	ID Image	d	Cream	Bosso
004001A	0.2	5	1	008001A	0.2	1	5	006006A	1.2	5	1	009001A	0.2	5	1
	0.7	5	1		0.7	3	3		3.2	5	1		0.7	5	1
	1.7	1	5		2.7	1	5		5.7	3	3		1.7	5	1
	3.2				3.2	3	3	009005A	0.2	5	1		3.7	3	3
005002A	0.2	5	1		5.2	5	1		3.2	5	1		5.2	5	1
	3.2	5	1	008004A	4.2	5	3		5.7	5	1	007001A	0.2	5	1
	5.7	5	1		5.7	3	3	006002A	0.7	5	1		2.7	5	1
				008007A	0.2	5	1		3.2	3	3		5.7	5	1
					3.2	3	3								

**Table 6:** Weighted average d values for filters.

Filter Type	Weighted Average d
Cream	2.89
Bosso	3.07

**Table 7:** Acquisition parameters and filters applied by anatomical region.

Anatomical Region	Energy (kVp)	Exposure (mAs)	Source-Image Distance (cm)	Applied Filters	Technical Observations
**Knee**	60–70	15–20	110–120	Cream (Exponential) + Bosso (Gaussian)	High attenuation: Compton effect predominates. Significant improvement in contrast and sharpness.
**Breast**	20–30	10–15	100–110	Mainly Bosso (Gaussian)	Low density; diffuse improvement. The Bosso filter reduces background noise efficiently.
**Wrist**	50–60	10–15	100–110	Cream (Exponential) + Bosso (Gaussian)	Like knee, but with less attenuation. The combination improves edge detail.

**Table 8:** Simulated results comparison.

Filter/Method	Contrast	PSNR (dB)	SSIM	SNR (dB)	Likert Value (Average)
Original	1.60	-	-	-	2.0
CLAHE	2.80	24.5	0.72	18.2	3.0
Unsharp Masking	2.60	23.1	0.68	17.9	2.8
Autoencoder (DAE)	3.10	28.3	0.81	20.4	4.1
CNN (U-Net)	3.40	29.5	0.87	21.2	4.5
Cream + Bosso (Proposed)	3.93	30.1	0.90	22.1	4.7

**Table 9:** Actual results per anatomical region (d = 1.4). Completed with Simulated PSNR, SSIM and SNR.

Anatomical Region	Filter	Optimal d	Contrast	Entropy	PSNR (dB)	SSIM	SNR (dB)	Likert Value (Mean)
**Knee**	Cream	1.4	2.05129	0.43618	30.1	0.90	22.1	4.9
	Bosso	1.4	2.05027	0.44029	29.4	0.88	21.2	2.5
**Breast**	Cream	1.4	2.6089	0.17977	28.1	0.86	20.3	3.2
	Bosso	1.4	2.7649	0.17977	28.5	0.87	20.6	3.2
**Wrist**	Cream	1.4	3.7580	0.22458	30.4	0.91	22.5	4.7
	Bosso	1.4	3.7874	0.22559	30.0	0.89	21.9	2.9

## Data Availability

The data presented in this study are available on request from the corresponding author due to restrictions (privacy, legal and ethical reasons, from ASOMEL Health Clinic).

## Abbreviations

The following abbreviations are used in this manuscript:

ANOVA	Analysis of Variance
SID	Source Image Distance
ROI	Region of Interest
PSNR	Peak Signal-to-Noise Ratio
CLAHE	Contrast-Limited Adaptive Histogram Equalization
DAE	Denoising Auto Encoder
IMSE	Inverse Mean Square Error
SNR	Signal-to-Noise Ratio
PSF	Point Spread Function
CNN	Convolutional Neural Network
U-Net	Convolutional Neural Network, designed for image segmentation task
PACS	Picture Archiving and Communication System
SSIM	Structural Similarity Index
